# Time-lapse imaging using dual-color coded quantitative differential phase contrast microscopy

**DOI:** 10.1117/1.JBO.27.5.056002

**Published:** 2022-05-16

**Authors:** Ying-Ju Chen, Yu-Zi Lin, Sunil Vyas, Tai-Horng Young, Yuan Luo

**Affiliations:** a National Taiwan University, Department of Biomedical Engineering, Taiwan; b National Taiwan University, Institute of Medical Device and Imaging, Taipei, Taiwan; c National Taiwan University, YongLin Institute of Health, Taipei, Taiwan

**Keywords:** phase contrast, quantitative phase imaging, time-lapse imaging

## Abstract

**Significance:**

Quantitative differential phase contrast (qDPC) microscopy enhances phase contrast by asymmetric illumination using partially coherent light and multiple intensity measurements. However, for live cell imaging, motion artifacts and image acquisition time are important issues. For live cell imaging, a large number of intensity measurements can limit the imaging quality and speed. The minimum number of intensity measurements in qDPC can greatly enhance performance for live imaging.

**Aim:**

To obtain high-contrast, isotropic qDPC images with two intensity measurements and perform time-lapse imaging of biological samples.

**Approach:**

Based on the color-coded design, a dual-color linear-gradient pupil is proposed to achieve isotropic phase contrast response with two intensity measurements. In our method, the purpose of designing a dual-color coded pupil is twofold: first, to obtain a linear amplitude gradient for asymmetric illumination, which is required to get a circular symmetry of transfer function, and second, to reduce the required number of frames for phase retrieval.

**Results:**

To demonstrate the imaging performance of our system, standard microlens arrays were used as samples. We performed time-lapse quantitative phase imaging of rat astrocytes under a low-oxygen environment. Detailed morphology and dynamic changes such as the apoptosis process and migration of cells were observed.

**Conclusions:**

It is shown that dual-color linear-gradient pupils in qDPC can outperform half-circle and vortex pupils, and isotropic phase transfer function can be achieved with only two-axis measurements. The reduced number of frames helps in achieving faster imaging speed as compared to the typical qDPC system. The imaging performance of our system is evaluated by time-lapse imaging of rat astrocytes. Different morphological changes in cells during their life cycle were observed in terms of quantitative phase change values.

## 1 Introduction

Optical microscopy plays an important role in material science, medical diagnosis, and biological research by acquiring informational and featureful images.^1^ Over the years, diverse imaging modalities have been introduced to improve the image contrast and resolution.^2^ The image contrast of regular label-free microscopy is induced directly from the light absorbance or scattering of the specimen.^3^^,^^4^ However, the translucent specimens, which are composed of weak phase features, barely absorb the illumination light and are thus difficult to be observed. Quantitative phase imaging (QPI) is a key technique for obtaining information from transparent specimens. It utilizes the phase shift generated by the specimen when light passes through the specimen.^5^

Unlike Zernike phase contrast microscopy^6^^–^^8^ and conventional differential interference contrast,^9^^,^^10^ which only offers qualitative phase contrast images, QPI retrieves quantitative phase information in terms of optical path-length difference of a specimen. It usually requires spatially coherent illumination (e.g., interferometry^11^^,^^12^), which creates speckle noise and limited spatial resolution. Partially coherent illumination offers unique advantages over coherent illumination, such as high lateral resolution and speckle noise-free images. A differential phase contrast (DPC) is an efficient method that can retrieve quantitative phase information by illuminating samples with partially coherent asymmetric patterns.^13^ Combining the concepts of pupil function engineering and computational postprocessing of acquired images, quantitative phase information can be extracted from the multiple intensity measurements. Various versions of qDPC have been proposed.^3^^,^^13^ The qDPC microscopy shows stable system performance and does not require the depth scanning that is essential in methods such as transport of intensity equation (TIE).^14^^,^^15^ In addition, qDPC can provide higher efficiency than Fourier ptychography.^16^ In qDPC, phase information can be directly separated and recovered from intensity through the weak object transfer function (WOTF) and the complementary pairwise illuminations.^3^^,^^9^ Similar to conducting Hilbert transforms from object space to the optical field,^17^ complex spectrum modulation can be realized by using a typical half circular amplitude mask.^3^^,^^9^ The technique reconstructs phase information by making pairwise measurements in vertical and horizontal directions.^18^ However, the resultant qDPC transfer function will not be circularly symmetric, which is essential for obtaining uniform phase images. Under partial coherent illumination, achieving isotropic qDPC transfer function with a minimum number of measurements is an important practical requirement for in vivo imaging of biological specimens.^9^^,^^19^^,^^20^ Various efforts to build up high-speed mechanisms to simplify qDPC systems have been proposed.^21^^,^^22^ With conventional monochromatic half-circle pupil, there is a trade-off between the isotropy of the phase transfer function and the number of required intensity images.^22^ Previously, various designs of monochromatic and color-coded pupil functions have been explored to acquire an isotropic phase transfer function with a minimum number of intensity measurements.^22^^,^^23^ One of the prominent methods is color encoding in pupil design, which can help in reducing the image acquisition time by obtaining the multicolor information at the same time. Although different combinations of the red, blue, and green colors are possible at the pupil plane, achieving isotropy of phase is difficult.^24^ It is observed that the shape of the pupil function plays a significant role in reducing the number of frames and isotropy of phase transfer function.

In this paper, we propose an approach that uses dual-color coded pupil engineering to achieve isotropic phase contrast response with two intensity measurements. In our method, the purpose of designing a dual-color coded pupil is twofold: first, to obtain a linear amplitude gradient for asymmetric illumination, which is required to get circular symmetry in transfer function, and second, to reduce the required number of frames for phase retrieval. Two-color images obtained from our systems are separated into different channels and processed separately to obtain the final image. The illumination is designed with a dual-color linear amplitude gradient to obtain an asymmetric illumination pattern. Compared to typical two-axis half-circle-measurements, the proposed masks significantly improve stability and accuracy of quantitative phase recovery. Due to color leakage between the different color channels of the image sensor, the captured images need calibration.^25^ Through pairwise images, the phase information can be retrieved from intensity measurements based on the WOTF. In our method, circular symmetry of the transfer function can be achieved with rather high-speed operation.^3^ In addition to improving the quality of the quantitative phase image, a reconstruction procedure including direct inverse function based on Tikhonov regularization has been applied to recover the phase with minimum imperfections.

The proposed method can perform 12× faster phase reconstruction than the conventional half-circle monochromatic pupil with the same image characteristics such as resolution and contrast. The reduced imaging speed can greatly help in monitoring developmental cell biological processes through time-lapse imaging. To understand the complex structures and morphology of rat astrocytes, time-lapse imaging of samples under a controlled oxygen environment was performed. Astrocytes are one type of glial cells that are dominant in the central nervous system and perform functions such as energy supplementation for neurons, blood–brain barrier regulation, glia scar formation, and glucose storage.^26^^,^^27^ This type of cell swells when brain ischemia occurs and leads to cerebral edema, which can be a possible target for therapy.^28^ To study cerebral edema arising from cell edema, an in vitro model was built by using the oxygen and glucose deprivation (OGD) method to culture astrocytes.^29^ Due to our label-free approach, the natural contents of the cells remain intact and the dynamic processes through the samples can readily be observed. With our method, the nature of the structural changes in the samples can be evaluated in terms of quantitative phase changes inside the acquired images. Our experimental results clearly demonstrated the capability of our system to perform time-lapse quantitative phase imaging.

## 2 Differential Phase Contrast Microscopy

### 2.1 System Configuration

**Fig. 1:**
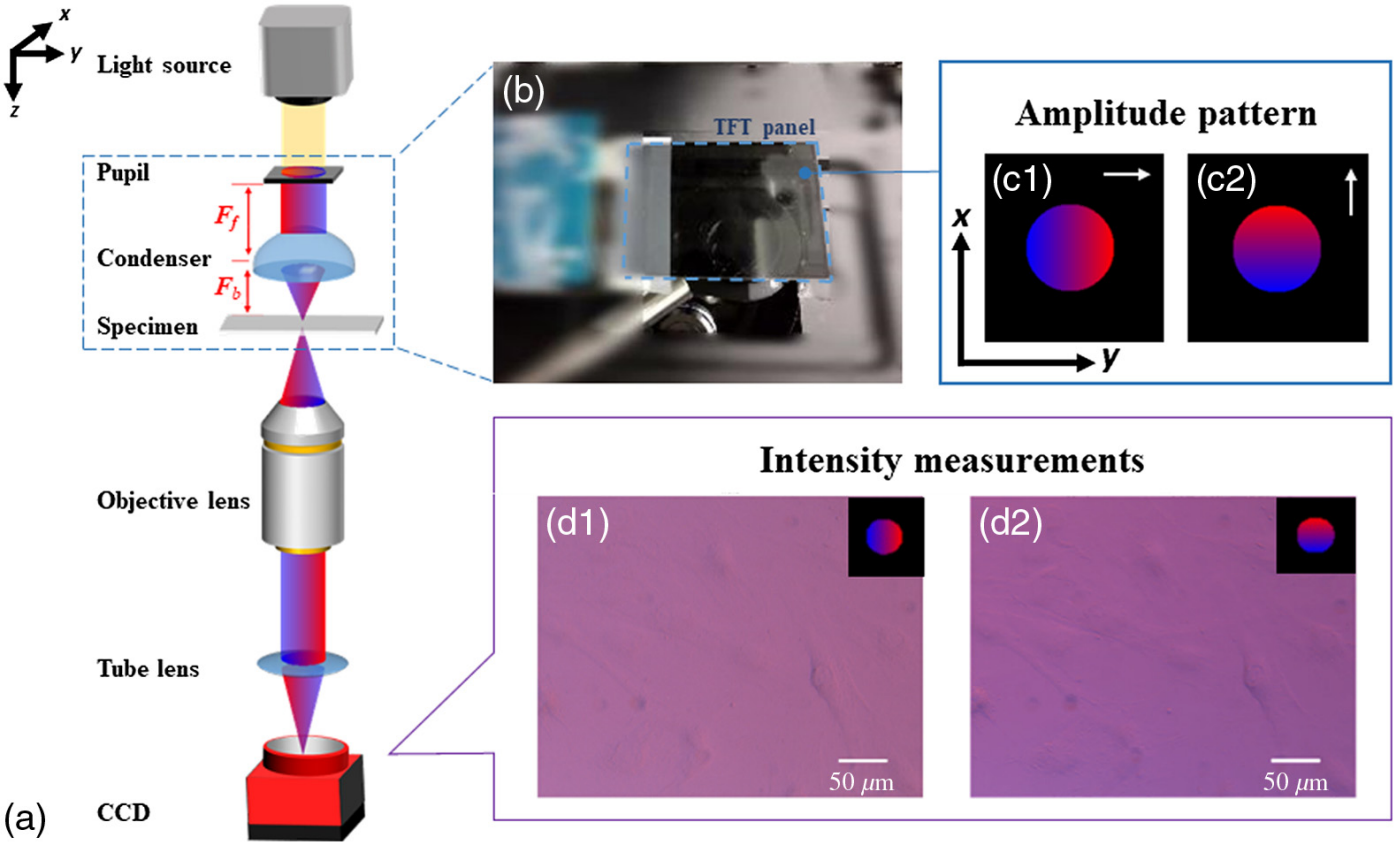
Schematic diagram of the proposed dual-color coded qDPC system. (a) Optical arrangement of qDPC microscope system. (b) Photograph of TFT panel. (c1) Dual-color linear-gradient amplitude masks along the horizontal direction. (c2) Dual-color linear-gradient amplitude masks along the vertical direction. (d1) and (d2) Raw intensity images obtained after projecting (c1) and (c2) masks.

The optical setup of the proposed dual-color coded qDPC system is shown in Fig. 1(a). On an inverted microscope (Olympus IX70), a programmable thin-film transistor (TFT) shield is placed on the Fourier plane of the condenser lens. The condenser lens is LA1951-ML from Thorlab. The front focal length of the condenser lens is 25.3 mm and the back focal length is 17.6 mm. The objective lens is LMPLN10XIR from Olympus, with a magnification value of 10 and a numerical aperture (NA) of 0.3. The focal length of the tube lens is f=180  mm (Olympus IX70). The TFT shield is a 2.8-in TFT Touch Shield v2.0 from Seed Studio. The pixel size is 18.2  μm and the resolution is 320×240. A photograph of the TFT panel is depicted in Fig. 1(b). Figures 1(c1) and 1(c2) show the dual-color coded linear-gradient amplitude mask along the horizontal and vertical directions, which are projected onto the TFT panel to obtain asymmetric illumination patterns for the samples. The TFT panel with the amplitude mask acts as a digital pupil, which allows dynamic control of illumination and enable minimal image acquisition time.

The intensity measurements are obtained by a color camera (Alvium 1800 u-500c) with the pixel size 2.2  μm, and the frame rate 67 fps. Figures 1(d1) and 1(d2) show the two raw intensity images after projecting linear color gradient pupils Figs. 1(c1) and 1(c2) onto the TFT panel. The two color masks are revealed as two different axes of masks produced by combing pairs of blue and red color linear-gradient pupils. We chose red and blue colors because the separation of the spectrum of red and blue sources is larger as compared to green, and hence the influence of the color leakage problem is expected to be less. The qDPC images are generated along different axes for quantitative phase reconstruction based on the WOTF after color leakage correction (CLC), which will be discussed in Sec. 2.2.

### 2.2 Color Leakage Correction

The dual-color coded amplitude mask displayed on the TFT shield filters incident white light into three color channels: red, green, and blue (R, G, and B). Each color channel carries different image information respectively. However, color spectra generated by the TFT shield may not thoroughly match the spectral response of the color camera and result in the overlapping of different color spectra on the camera sensor. Due to the color leakage, the light intensity of each TFT channel influences adjacent channels and severely degrades reconstructed image quality.^25^ The RGB-light intensities, collected by the color camera, can be formulated as follows:

$$[\begin{array}{c} I_{\mathrm{Camera}}^{R}\\ I_{\mathrm{Camera}}^{G}\\ I_{\mathrm{Camera}}^{B} \end{array}]=T[\begin{array}{c} I_{\mathrm{TFT}}^{R}\\ I_{\mathrm{TFT}}^{G}\\ I_{\mathrm{TFT}}^{B} \end{array}],$$ (1)

where $I_{\mathrm{Camera}}^{R}$ and $I_{\mathrm{TFT}}^{R}$ are the intensity of light and the spectra, which are decided by the *R* channel of the camera and TFT shield. T is a 3×3 matrix that transfers the intensity of the original spectrum on the TFT shield to the intensity received by the camera. The transfer matrix T is given by^25^

$$T=[\begin{array}{ccc} L_{R}^{R} & L_{R}^{G} & L_{R}^{B}\\ L_{G}^{R} & L_{G}^{G} & L_{G}^{B}\\ L_{B}^{R} & L_{B}^{G} & L_{B}^{B} \end{array}],$$ (2)

where $L_{w}^{v}$ is the leakage ratio from the v channel of the TFT shield to the w channel of the camera. The v channels of the TFT shield and the w channels of the camera both include R, G, and B channels. $L_{w}^{v}$ can be obtained through experimental measurements. To recover the original images before light passes through the filters of the camera, the inverse matrix of T is multiplied with the intensity received from the color camera and given by

$$[\begin{array}{c} I_{\mathrm{TFT}}^{R}\\ I_{\mathrm{TFT}}^{G}\\ I_{\mathrm{TFT}}^{B} \end{array}]=T^{-1}[\begin{array}{c} I_{\mathrm{Camera}}^{R}\\ I_{\mathrm{Camera}}^{G}\\ I_{\mathrm{Camera}}^{B} \end{array}].$$ (3)

By the virtue of CLC, the quality of reconstructed images will be significantly improved. Nevertheless, every color camera has a different spectral response, T and $T^{-1}$ will differ for different color cameras, and initially, every system has to measure the matrix T to mitigate color leakage errors.

**Fig. 2:**
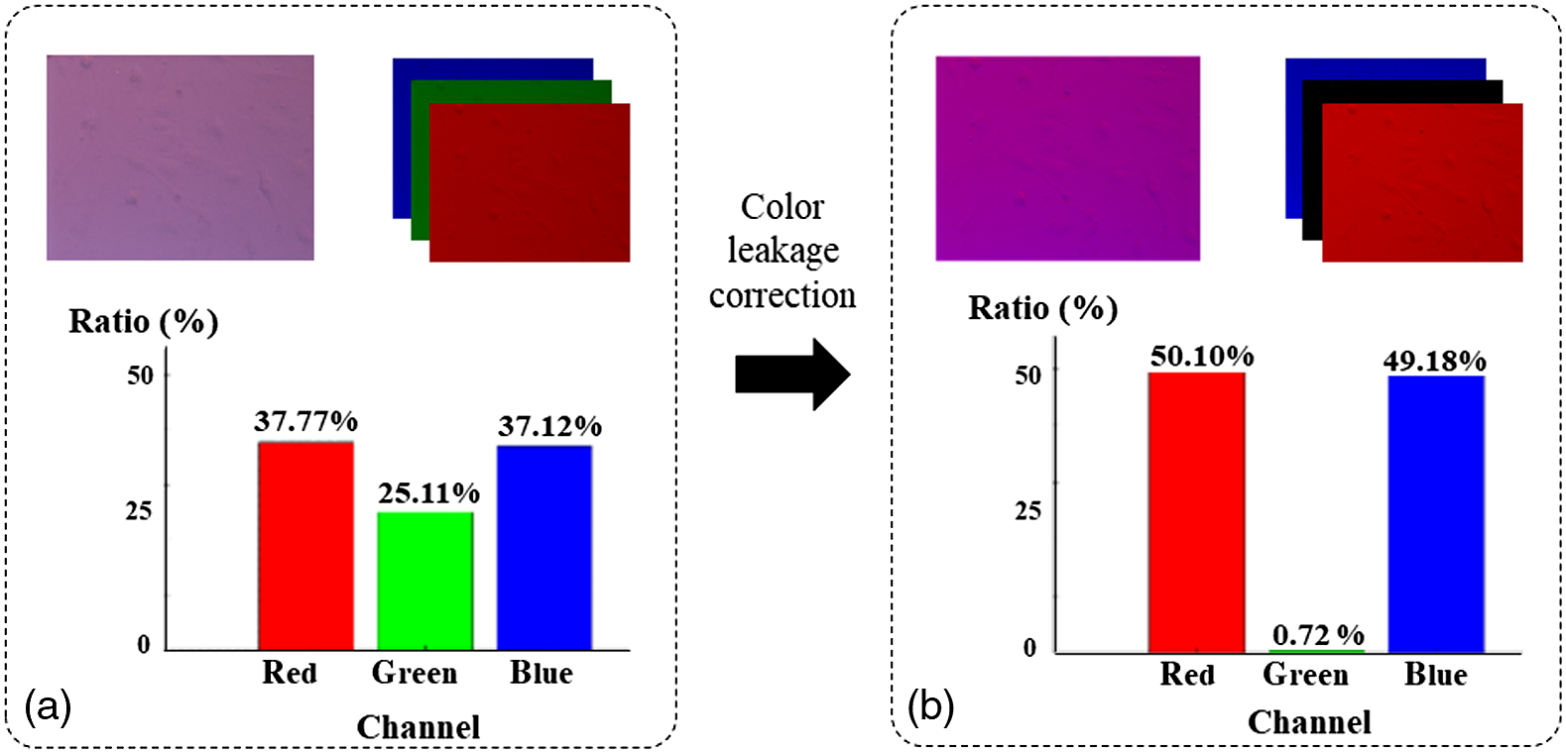
The intensity ratio between different color channels before and after CLC. (a) An uncorrected image and corresponding bar chart for intensity ratio between different channels. Uncorrected image is separated into three channels to measure the cross-talk phenomena between different colors. (b) Correct image after applying CLC and corresponding bar chart.

Figure 2 shows the results of CLC by calculating the ratio of intensity of three channels. The image of the specimen illuminated under a dual-color coded gradient pupil was separated into three channels. Figure 2(a) shows the uncorrected image and corresponding bar chart for the intensity ratio between different channels. The color leakage value of the green signal is around 25.11% under red and blue light illumination. Figure 2(b) shows the results after applying the CLC. The effectiveness of CLC can be easily observed as changes in the appearance of the RGB color, as shown in Fig. 2(b). The intensity ratio of the green signal is reduced to 0.72% while the red and blue signals are both around 50%, which mitigates the leakage due to mismatch spectrum between illumination and detector. The experimental results indicate that the color leakage algorithm corrects each color channel significantly.

### 2.3 Differential Phase Contrast Transfer Function

To treat a specimen as a weak phase object, we can utilize the phase transmission function $O(r)=e^{-\alpha (r)}e^{i\phi (r)}$, where r denotes the spatial coordinates (x,y), α(r) and ϕ(r) indicate attenuation factor and optical phase, respectively.^30^ To implement the weak object approximation, either phase object should have a small phase delay or be embedded in a uniform substrate with a constant refractive index to fulfill the slowly varying phase (i.e*.,* refractive index condition).^18^^,^^31^ The working principle of our microscope can be described by a 4f optical system as shown in Fig. 1. The amplitude mask of the condenser pupil is defined as $S(u)=V_{\lambda_{i},j}(u)\mathrm{circ}(u)$ on the TFT shield. $u=(u_{x},u_{y})$ represents spatial frequency coordinates. $V_{\lambda_{i},j}(u)$ is the amplitude mask function, and i denotes the number of working wavelengths. In our case, i=1,2, and j decide the orientation of linear distribution along x (j=1) or y (j=2) axis. $\rho_{c}={\mathrm{NA}}_{\mathrm{condenser}}/\lambda$, where ${\mathrm{NA}}_{\mathrm{condenser}}$ is the numerical aperture of the condenser and λ is the working wavelength of the light source. In our approach, this function generates two linear amplitude gradient patterns that compensate for each other under different wavelengths. $V_{\lambda_{i},j}(u)$ and circ(u) can be defined as^22^

$$v_{\lambda_{i},j}(u)=\{\begin{array}{l} \frac{(-1{)}^{i-1}u_{x}+\rho_{c}}{2\rho_{c}},\;j=1\\ \frac{(-1{)}^{i-1}u_{y}+\rho_{c}}{2\rho_{c}},\;j=2 \end{array},$$ (4)

$$\mathrm{circ}(u)=\{\begin{array}{c} 1,\;|\frac{u}{\rho_{c}}|\leq 1\\ 0,\;|\frac{u}{\rho_{c}}|>1\; \end{array}\;.$$ (5)

P(u) is the pupil function in the objective lens, and under partially coherent illumination conditions, the resultant image intensity captured on the camera can be written as

$$I(r)=\int \int |F\{F[\sqrt{S(u)}e^{i2\pi ur}W(r)]\cdot P(u)\}{|}^{2}d^{2}u,$$ (6)

where F denotes the Fourier transform induced by a condenser lens or objective lens. The outmost integration with respect to u represents incoherent supposition of scattered light due to each point source S(u) at the image plane. Invoking a weak object approximation with Taylor expansion, we can linearize the translucent object as W(r)=1−α(r)+iϕ(r). The intensity of a weak phase specimen in the Fourier space can then be written as

$$\overset{˜}{I}(u)={\overset{˜}{H}}_{\mathrm{phase}}\cdot \overset{˜}{\phi }(u)+{\overset{˜}{H}}_{\mathrm{amp}}\cdot \overset{˜}{\alpha }(u)+{\overset{˜}{H}}_{b}\cdot \delta (u)$$ (7)

where $\tilde{\phi }(u)$ and $\overset{˜}{\alpha }(u)$ represent the phase and absorption of the specimen, respectively. Hence, the expressions for the phase transfer function ${\tilde{H}}_{\mathrm{phase}}$, amplitude transfer function ${\tilde{H}}_{\mathrm{amp}}$, and background term ${\tilde{H}}_{b}$ are given by^3^

$${\overset{˜}{H}}_{\mathrm{phase}}=j[\iint S(u^{\prime })P(u^{\prime }+u)P^{*}(u^{\prime })d^{2}u^{\prime }-\iint S(u^{\prime })P^{*}(u^{\prime })P(u^{\prime }-u)d^{2}u],$$ (8)

$${\overset{˜}{H}}_{\mathrm{amp}}=\iint S(u^{\prime })P(u^{\prime })P^{*}(u^{\prime }-u)d^{2}u^{\prime }-\iint S(u^{\prime })P(u^{\prime }+u)P^{*}(u^{\prime })d^{2}u^{\prime },$$ (9)

$${\overset{˜}{H}}_{b}=\iint S(u^{\prime })|P(u^{\prime }){|}^{2}d^{2}u^{\prime }.$$ (10)

Furthermore, in our approach, isotropic DPC imaging is achieved using dual-color illumination in conventional DPC imaging. By adopting dual-color linear-gradient amplitude masks, isotropic DPC images are obtained. The amplitude mask acts like a wavelength filter where the two colors provide complementary gradient vectors to each other. The dual-color coded qDPC images ($I_{\mathrm{DPC},k}$) can be obtained as

$$I_{\mathrm{DPC},k}=(I_{\lambda_{1},k}-I_{\lambda_{2},k})/(I_{\lambda_{1},k}+I_{\lambda_{2},k}).$$ (11)

Following this step, for an aberration-free system, ${\overset{˜}{H}}_{\mathrm{amp}}\cdot \overset{˜}{\alpha }(u)$ and ${\overset{˜}{H}}_{b}\cdot \delta (u)$, are eliminated since these two terms are symmetrical. Hence, only the phase term ${\overset{˜}{H}}_{\mathrm{phase}}\cdot \overset{˜}{\phi }(u)$ is retained, which represents the relationship as${\overset{˜}{I}}_{\mathrm{DPC}}(u)={\overset{˜}{H}}_{\mathrm{phase}}\cdot \overset{˜}{\phi }(u)$. Based on Eqs. (4)–(6), dual-color linear-gradient amplitude masks are designed in blue and red, which correspond to two different axes of measurements to achieve isotropic transfer function. The axis of the color gradient is represented by arrows as shown in Fig. 1. Experimentally, we extract the intensity information ($I_{\lambda_{1},k},I_{\lambda_{2},k}$) from the image captured by the camera within the blue and red channels (k=1- to 2-axis). In our case, $\lambda_{1}$ is red, and $\lambda_{2}$ is blue as shown in Fig. 1. Quantitative phase information can then be obtained by solving equation Eq. (9) (amplitude transfer function) using Tikhonov regularization and given by^3^

$$\overset{˜}{\phi }(r)=F^{-1}\{\frac{\sum_{i}({\overset{˜}{H}}_{\mathrm{phase},i}\;\cdot \;F(I_{\mathrm{DPC},i}(r)))}{\sum_{i}|{\overset{˜}{H}}_{\mathrm{phase},i}{|}^{2}+\gamma }\},$$ (12)

where γ represents the regularization parameter whose typical values we choose in our phase image reconstruction to be approximately $10^{-3}$ to $10^{-4}$. Similarly, absorption transfer can be evaluated.^24^^,^^32^

## 3 Simulation Results

**Fig. 3:**
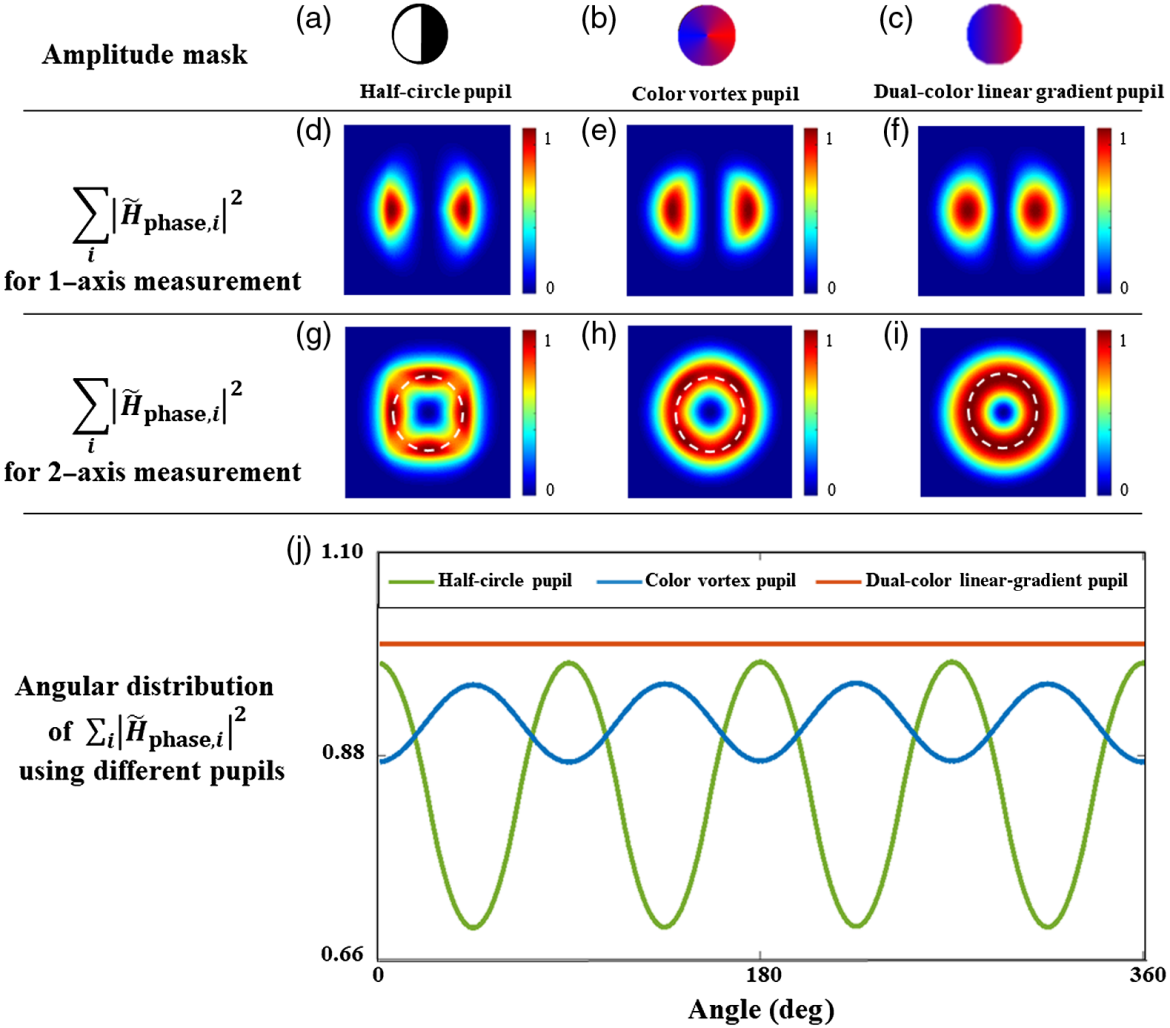
(a)–(c) Comparison of different simulation results based on different amplitude masks: (a) half-circle pupil, (b) two-color vortex pupil, and (c) dual-color linear-gradient pupil. (d)–(f) Phase transfer functions along one axis. (g)–(i) Phase transfer functions along two-axes. (j) Comparison of the angular distribution of the phase transfer function for the three pupil masks. The phase profiles are drawn along the dotted lines in (g)–(i).

To compare the performance of the dual-color coded qDPC with previously proposed methods, simulations of the phase transfer function ($\sum_{i}|{\tilde{H}}_{\mathrm{phase},i}{|}^{2}$) for multiaxis measurements are performed. The phase transfer function of our method is compared with the half-circle pupil^3^ and color vortex pupil.^33^ Figures 3(d)–3(f) are the respective normalized simulation results of the phase transfer function based on the one-axis approach, while Figures 3(g)–3(i) are results based on the two-axis approach. The measurement axis i=1 denotes two masks with opposite directions of the gradient along the horizontal direction and i=2 denotes two masks with opposite directions of the gradient along the vertical direction. The pairwise one-axis image is from the red and blue channels in one measurement. As the resultant qDPC transfer function of the one-axis approach is unable to thoroughly recover phase in all the directions, multiple axes measurements are essential to achieve the isotropy of transfer function which makes the system ideally shift-invariant. We can see from Fig. 3(i) that the isotropic transfer function can be realized with just fewer measurements with the proposed dual-color linear-gradient pupils. The phase transfer function along the one-axis measurement is shown in Figs. 3(d)–3(f). Similarly, the phase transfer functions for two-axis measurements are shown in Figs. 3(g)–3(i). Figure 3(j) shows the comparison of the angular distribution of the phase transfer function $\sum_{i}|{\tilde{H}}_{\mathrm{phase},i}{|}^{2}$ according to the white dotted circles drawn on Figs. 3(g)–3(i) on the same set of axes. From Fig. 3, it is clear that dual-color linear-gradient pupils can show nearly constant frequency along the circular region among the three different pupils, which indicates that dual-color linear-gradient pupils can realize a better isotropic qDPC transfer function than the typical half-circle pupil and the color vortex pupil.^33^

## 4 Performance Evaluation of Dual-Color Coded qDPC Microscopy

To verify the quantitative measurement capability and accuracy of the developed dual-color coded qDPC microscope, a microlens array thickness of 862 nm was used to demonstrate phase gradient images along the vertical and horizontal axis. The microlens array surrounded by air (refractive index $n_{s}=1$) has the refractive index $n_{b}=1.46$.

**Fig. 4:**
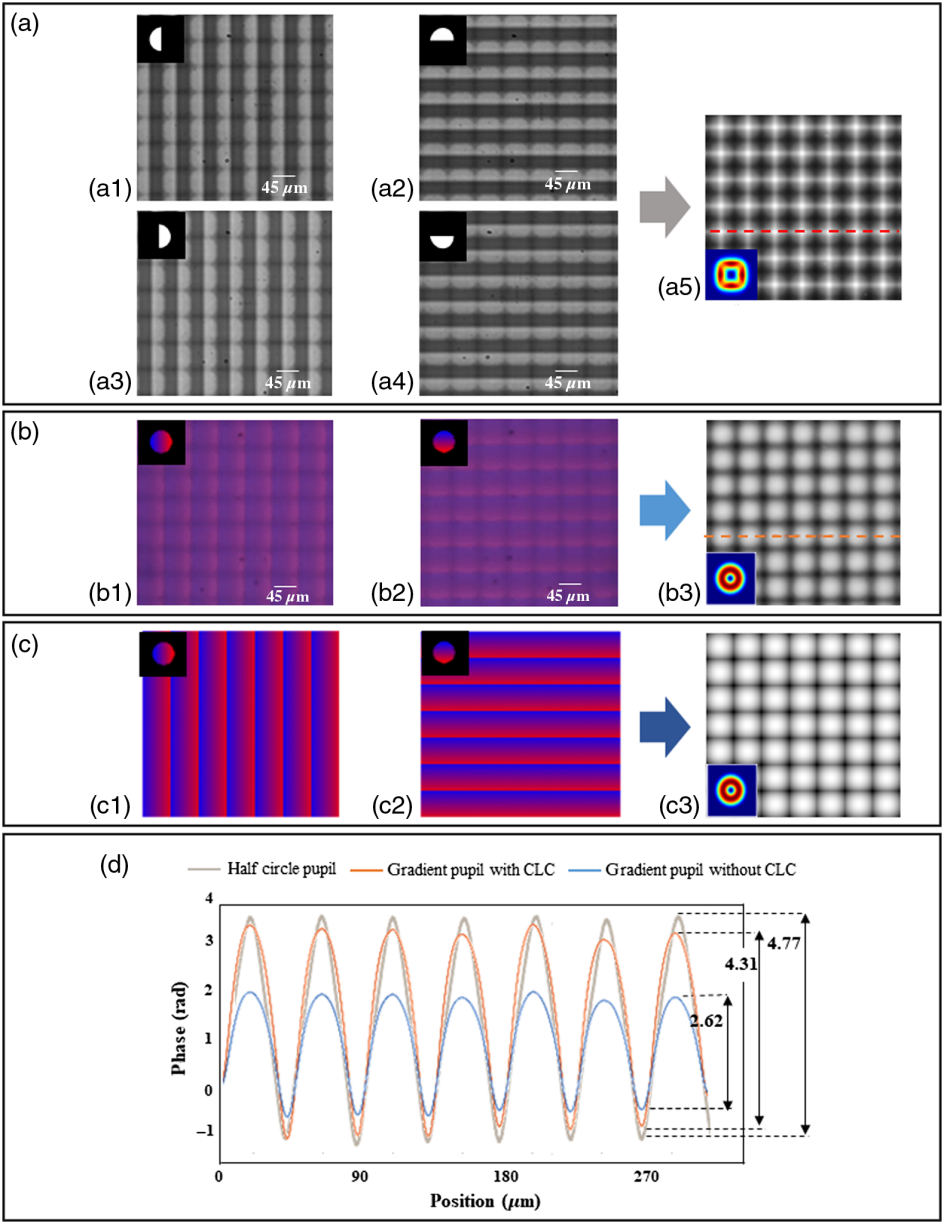
(a) Experimental results of quantitative phase measurement of a microlens array by using a half-circle pupil: (a1)–(a4) raw images for different orientations of the half-circle pupil and (a5) reconstructed quantitative phase images with phase transfer function in the inset. (b) Experimental results of quantitative phase measurement of a microlens array by using dual-color linear-gradient pupils: (b1) and (b2) raw images of the dual-color linear-gradient in various orientations and (b3) reconstructed quantitative phase images with phase transfer function in the inset. (c) Simulation results of quantitative phase measurement of a microlens array by using dual-color linear-gradient pupils: (c1) and (c2) raw images for different orientations of the dual-color linear-gradient and (c3) reconstructed quantitative phase images and phase transfer function in the inset. (d) Phase profile along the dashed line in (a5) and (b3) images with and without CLCs.

Experimental results of the quantitative phase measurement of a microlens array by conventional half-circle pupil (two-axis measurements) and the dual-color linear-gradient pupil are shown in Fig. 4. Figures 4(a1)–4(a4) show the raw images of the microlens array obtained for different orientations of the pupil. Reconstructed quantitative phase images of microlenses are shown in Fig. 4(a5) with the phase transfer function in the inset. Due to few angle measurements with a half-circle pupil, the phase transfer function is anisotropic, and the resultant phase image is not isotopically reconstructed. The raw images of a microlens array obtained by the dual-color linear-gradient pupil are shown in Figs. 4(b1) and 4(b2). Corresponding quantitative phase reconstructed images of microlenses are shown in Fig. 4(b3). With these results, it is evident that dual-color linear-gradient pupil illumination can provide isotropic phase reconstruction in a few measurements. Figure 4(c) shows simulation results of the microlens array using dual-color linear-gradient pupil. A comparison between with and without the CLC is shown in Fig. 4(d). The blue and red lines indicate the results before and after being applied CLC, respectively. From Fig. 4(d), we can see the effect of CLC in improving the image quality. There is a difference of 39% between the two phase distributions. The estimated phase is 4.53 rad is obtained as

$$\Delta Ø=\frac{2\pi D(n_{b}-n_{s})}{\lambda }.$$ (13)

The difference between the experimentally measured phase values after CLC is around 4.31 rad. The deviation in phase values may be due to uncertainties in the size of each microlens. The deviation with respect to estimated values is around 4.8%. The equivalent thickness of the sample is around 0.82  μm. These results show the accuracy of our method is around 95%.

**Fig. 5:**
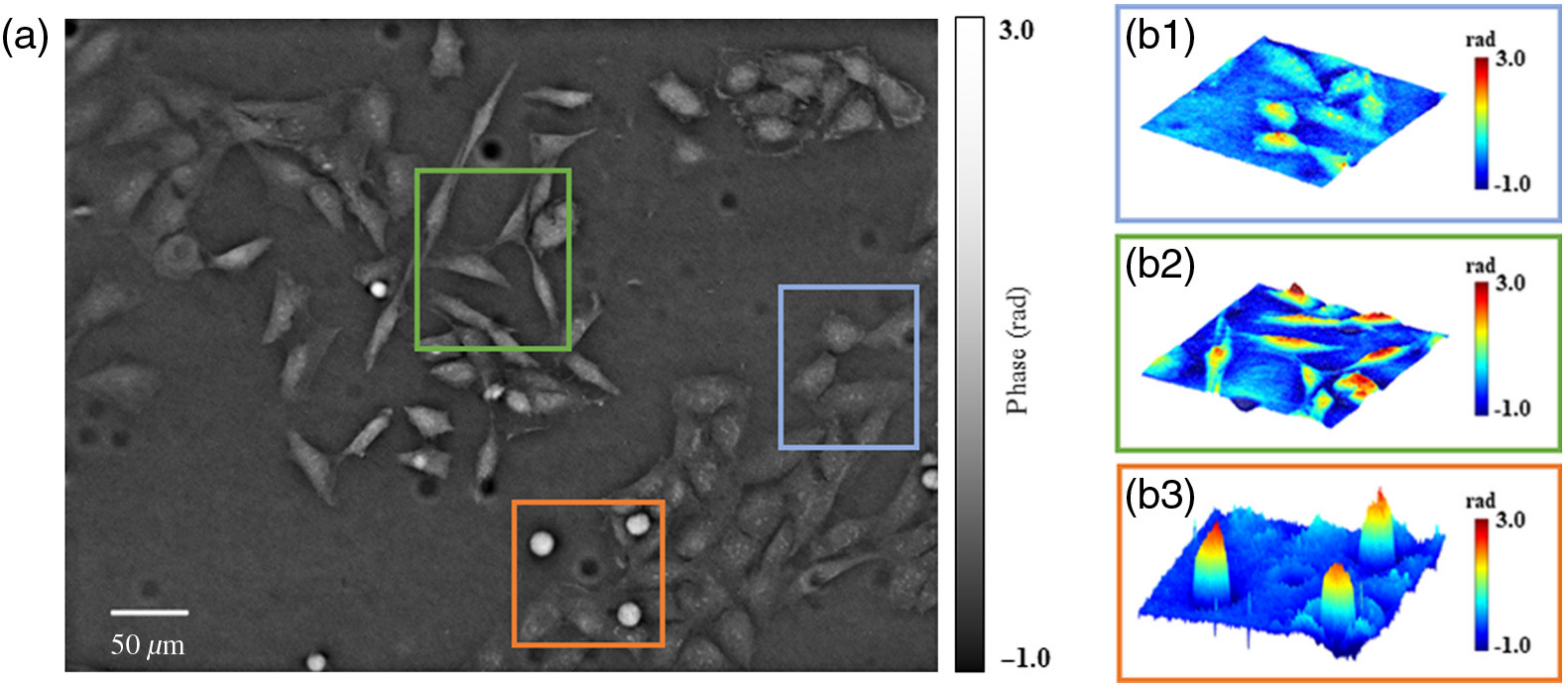
Experimental demonstration of qDPC imaging of live rat astrocytes. (a) Recovered phase images using dual-color coded qDPC with 10×0.3 NA objective lens. (b1)–(b3) 3D views of the zoomed-in parts shown in the solid box region in Figure (a) to demonstrate the different morphologies of the cells.

## 5 Live Cell Imaging Using Dual-Color Coded qDPC Microscopy

We further demonstrate the performance of our system by imaging label-free rat astrocytes. The retrieved phase image of the cells is shown in Fig. 5(a). Figures 5(b1)–5(b3) depict zoomed-in three-dimensional (3D) views of various parts of the specimen. Different morphologies of cells can easily be observed in these figures. Figure 5(b1) shows an astrocyte that looks flat and expanded. Slender dendrites of astrocytes can be clearly seen in Fig. 5(b2). Apoptosis is a programmed death that is actively performed by cells. The apoptosis can also be observed easily through the images obtained from our system. Figure 5(b3) shows the apoptosis of cells inside the specimen.

**Fig. 6:**
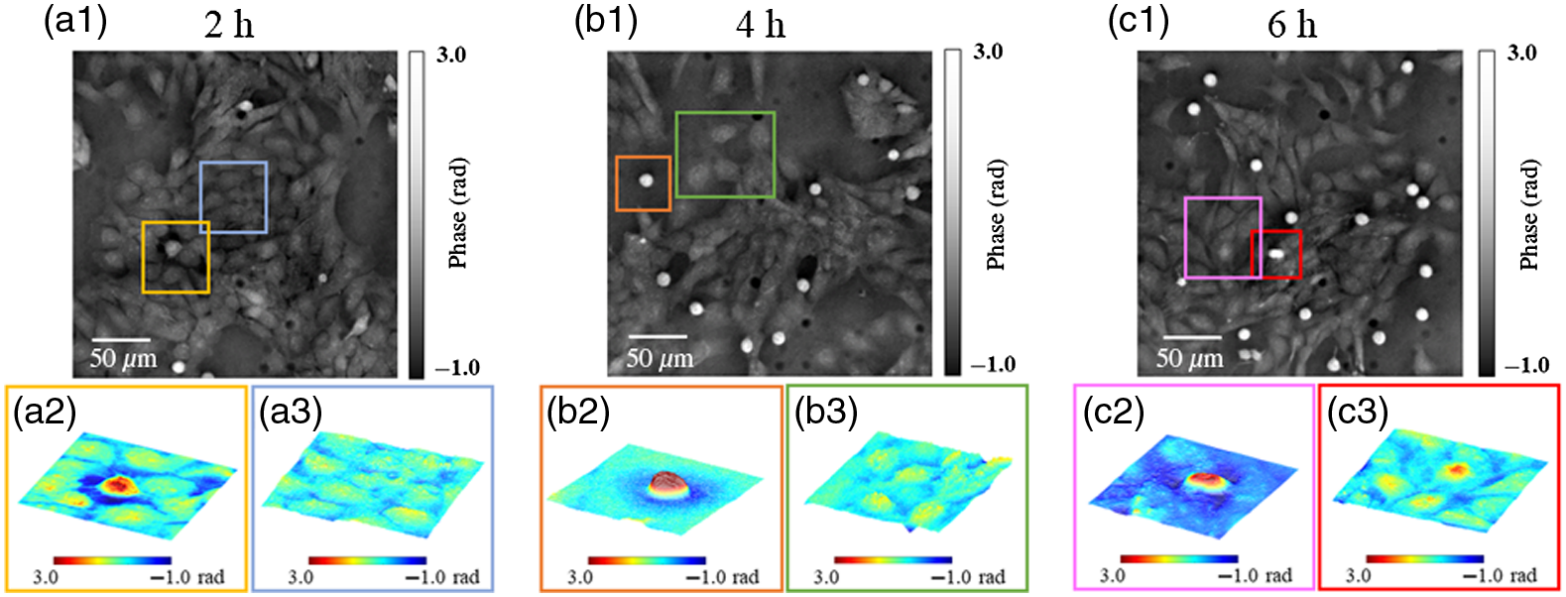
The phase images of rat astrocyte captured at three different times. (a1), (b1), and (c1) Recovered phase images of astrocyte obtained at 2, 4, and 6 h, respectively. (a2, a3), (b2, b3), and (c2, c3) Zoomed-in 3D view of corresponding portions marked with colored boxes.

The dual-color coded qDPC was used to observe the morphology of rat astrocytes under a low-oxygen environment. To see the influence of low-oxygen concentration, the cells were measured after 2, 4, and 6 h, and results are shown in Fig. 6. The corresponding zoomed-in images (marked with colored boxes) at different locations of the specimens are shown at the bottom of Fig. 6. During the cell growth process, the astrocyte becomes flat and expands with a broad leading edge, whereas few dendrites move around inside the culture dish. Figure 6 shows a series of qDPC images by using a dual-color linear-gradient pupil. The details of the apoptosis process of cells can be observed in Figs. 6(a2) and 6(c2). In response to environmental stimuli, cells undergo self-determined death after receiving a specific signal. While the cells shrink, their phase difference value gradually increases from 1.5 to 2.5 rad. As expected, the number of dead cells increased with time. Due to low-oxygen concentration, the mean phase of cells increased from ∼1.8 to ∼2.0  rad. Figures 6(a3), 6(b3), and 6(c3) show the flat cells with increased phase values due to OGD. Although the environment causes swelling of astrocytes, the cells after 6 h still show a division process, which is shown in Fig. 6(c2). The quantitative phase difference values are indicated by grayscale. These results show the capability of the dual-color coded qDPC system for cell biological studies. The reduced number of experimental measurements with isotropic phase reconstruction offered by our method will greatly help the biological studies, which require multiple transient images and long-term monitoring of specimens.

When white light propagates into dielectric media, the speed of different wavelength components travels at different speeds, which results in chromatic dispersion. There are several methods to perform the analysis of the dispersion from the sample. Chromatic dispersion of an imaging system can be used to obtain phase directly. One such method is the TIE, which can accurately reconstruct the quantitative phase.^34^ The other possible approach to study the dispersive properties of a sample is through label-free hyperspectral imaging, in which both topographic and spectral information can be studied at once.^35^ The acousto-optic filtration of light in a lens-in-lens common-path interferometer can also be used to obtain quantitative phase information for multiple wavelengths.^36^ In addition, a diffraction phase microscopy has also been applied for the quantitative dispersion measurement.^37^ In our study, we consider objects nondispersive and we have not performed any spectral analysis of the sample together with the quantitative phase measurements. It will require a separate theoretical framework to correlate the illumination color with the dispersion from the sample and its relationship with the phase transfer function. Furthermore, validating it experimentally will be another challenge. In our work, we used partially spatially coherent light, and spectral and coherence effects have not been studied. Absorbance and scattering coefficients of biological samples with different transmittance can be measured using techniques such as cavity ring-down spectroscopy.^38^ Previous studies assessed the absorption and phase of the samples for various colors.^32^ In our study, the phase-retrieval algorithm is presented based on a WOTF. Our phase estimate is based on a computational model that assumes weak scattering of objects. Following previous studies, we considered transparent cellular specimens for which the WOTF approximation holds true. Due to its transparent nature, we have not considered the absorption of light by the sample.

## 6 Conclusion

It is shown that dual-color linear-gradient pupils in qDPC can outperform half-circle and vortex pupils, and the isotropic phase transfer function can be achieved with only two-axis measurements. The limitation of the time-consuming multiple axis measurement and reconstruction artifacts caused by missing frequencies in a half-circle pupil can be overcome with the dual-color linear-gradient pupil. The reduced number of frames helps in achieving faster imaging speed as compared to the typical qDPC system. The imaging performance of our system is evaluated by time-lapse imaging of rat astrocytes. Different morphological changes in cells during their life cycle were observed in terms of quantitative phase change values. Present studies show the potential of the dual-color coded qDPC system for quantitative biomedical imaging for cell research.
